# Book Notices

**Published:** 1895-01

**Authors:** 


### Book Notices.

MEDICAL JURISPRUDENCE, FORENSIC MEDICINE, AND TOXICOLOGY. By R. A. Witthaus, A. M., M. D., Professor of Chemistry, Physics, and Hygiene in the University of the City of New York, etc., etc., and Tracy C. Becker, A. B., LL. B., Counselor-at-Law and Professor of Criminal Law and Medical Jurisprudence in the University of Buffalo. In four volumes. Volume II. Large 8vo, 751 pages, illustrated with wood-cuts and three plates, two of them in colors. Price, in muslin, $5.00; in brown sheep and in law style, $6.00 per volume. Sold by subscription only. William Wood & Company, Publishers, 43, 45, 47 East Tenth Street, New York City.

In the preparation of this volume the same general rule has been followed as in the one that preceded it.

The opening chapter is by Prof. Edward S. Wood, M. D., of Harvard University, on "Examination of Blood and Other Stains." Prof. Wood devotes eighty pages to the consideration of this subject, giving in detail the composition and properties of blood, the methods employed, chemical, spectroscopic, microscopic, etc., for the identification of blood stains. Menstrual stains, lochial stains, nasal blood stains, and seminal stains, are given due consideration by him.

The second chapter is on "The Examination of Hair," by the same writer; and the sub-headings are distinction between human and animal hairs, distinctions between hairs from different portions of the body, from what individual did the hairs in question originate, and were the hairs in question pulled out, or did they fall out spontaneously?

Chapter III was written by Prof. J. Chalmers Cameron, M. D., of McGill University, Montreal, on "Abortion and Infanticide." Fifty pages are taken up in the discussion of abortion, and eighty-two pages in that of infanticide. Everything, it seems, that would throw any light on these subjects, in a medico-legal sense, is here given. In considering the subject of abortion, the writer states that "four main questions suggest themselves: 1. Has abortion taken place? 2. If so, was it *spontaneous* (from natural causes), or induced (by the intentional act of the mother or any other person)? 3. If intentionally induced, was the act justifiable or criminal? 4. Did the induced abortion injure health or destroy life?"

Criminal abortion, measures used to produce abortion, sequelæ of abortions, the duties of medical experts in cases of criminal abortion, and illustrative cases, are the subdivisions made by Dr. Cameron in his discussion of the subject. The subject of infanticide has received from the learned doctor the consideration that its importance demands. Plain rules are laid down for the guidance of the physician in determining the viability of the child, the probable length of time it lived after birth, whether death was due to natural causes, or to violence, violent causes of death, and methods of performing a post mortem examination upon a new-born child for medico-legal purposes.

"Determination of Survivorship," by Tracy C. Becker, LL. B., and John Parmenter, M. D., is the subject of the fourth chapter of this volume.

Chapter V is devoted to the subject of "When Medical Examination or other Inspection of living human body is permitted or required by Courts of Law; and is written by Tracy C. Becker LL. B.

"Pregnancy, Labor, and the Puerperal State," by J. Clifton Edgar, M. D., is the subject of the sixth chapter, and these subjects receive careful attention at his hands under the following sub-headings:

Average duration of pregnancy and protracted gestation; relation between menstruation and impregnation; diminished duration of pregnancy viability; live birth; resemblances—mother marks; substitution of children; supposititious children; disputed pregnancy, labor, and the lying-in state; the symptoms and signs of pregnancy in the living; feigned pregnancy; feigned labor; feigned lying-in state; signs of recent delivery in the living; signs of recent delivery in the dead; corpus luteum; superfœtation; the period that must elapse after delivery before the woman can again become pregnant; unconscious impregnation; pregnancy and delivery; post-mortem parturition; and can the fœtus live after the death of the mother?

Chapter VII is on "Sexual Incapacity in its Medico-legal Relations." This chapter was written by Irving C. Rosse, M. D., and is divided into the following sub-headings:

Impotence and sterility; disputed sexual capacity and paternity; impotence and bastardy; sexual incapacity and adoption; sexual incapacity as it concerns the dissolution of a valid marriage; relative sterility; genesis incapacity in criminal affairs; the plea of impotence in accusations of unchaste conduct; the causes of sterility and impotence; impotence and sterility in women; sterility from congenital and other defects; pathological conditions of sterility; impotence in man; and influencing conditions arising from accidents and disease.

The next chapter is on the subject of "Medico-Legal Considerations of Rape," and is written by J. Clifton Edgar, M. D., and James C. Johnston, M. D. This chapter will be of great interest to Southern physicians and lawyers who come in contact with the colored rape fiend and his outraged victims.

"Unnatural Crimes," by Irving C. Rosse, M. D., is the subject of the next chapter. The subjects of sexual inversion, beastiality; pederasty, tribodism, irrumation and fellation are discussed in this chapter.

"Railway Injuries, their Clinical and Medico-Legal Features," is the subject of the next chapter. This chapter was written by W. B. Outten, M. D., and is really an exhaustive monograph by one of the leading railway surgeons of this country, and in it the various railway accidents and resultant injuries are discussed in detail.

The volume is completed by a well written chapter on "Simulated Diseases," by W. Thornton Parker, M. D. H.

---

A Primer of Psychology and Mental Disease. By C. B. Burr, M. D., Medical Superintendent of the Eastern Michigan Asylum. Member of the American Medico-Psychological Association, the State Medical Society, the Pontiac Medical Society. Corresponding Member of the Detroit Medical and Library Association. Price, in cloth, $1.00. George S. Davis, Publisher, Detroit, Mich. 1894.

This little book containing 84 pages of reading matter is divided into three parts.

Part first treats of psychology, and is a very clear, concise statement of the essentials of this science, and prepares the reader for—

Part second, which treats of the various forms of insanity. To the general practitioner not skilled in the diagnosis of mental diseases, this portion of the book will be especially helpful in enabling him to determine the form of insanity with which he has to deal, and the probability of recovery.

Part third discusses the management of insanity.

After speaking of requisites to be found in the Medical Attendant the author treats of administration of food, medicine, personal attention and nursing, exercise, employment and diversion, correcting pernicious habits and checking morbid impulses, manual and mechanical restraint, etc. The author nowhere speaks of the kinds of drugs to be used. This is beyond the scope of the work.

It is an excellent book for those who have time to read only a primer. F.

---

THE CARE AND FEEDING OF CHILDREN. A Catechism for the use of Mothers, and Children's Nurses. By L. Emmett Holt, M. D., Professor of Diseases of Children in the New York Polyclinic; Attending Physician to the Babies' Hospital and the Nursery and Child's Hospital, New York. Sixty-six pages. Printed on fine heavy paper. Cloth cover. Price 50 cents. D. Appleton & Co., Publishers, New York City. 1894.

If a copy of this little book could be placed in the hands of every mother and child's nurse in this country, it would be the means of preventing much suffering and the saving of many lives. The vast majority of the diseases of early childhood are caused by improper feeding, and usually as the result of ignorance in this matter on the part of the mother. Dr. Holt has, in this book, laid down rules for their guidance that can be easily understood, and the possessor of a copy of it would have no difficulty in feeding the baby the proper food at the right time and in proper quantities.

We heartily commend the book to the consideration of every physician who is called on to treat digestive disorders of infancy and childhood. H.

---

A HAND-BOOK OF MEDICAL MICROSCOPY FOR STUDENTS AND GENERAL PRACTITIONERS, including Chapters on Bacteriology, Neoplasms, and Urinary Examinations. By James E. Reeves, M. D., Member of the Association of American Physicians; Ex-President of the American Public Health Association; Ex-Member and Secretary of the State Board of Health of West Virginia; Ex-President of the State Medical

Society of West Virginia; Author of a "Practical Treatise on Enteric or Typhoid Fever," etc., etc., etc. With a glossary and Numerous Illustrations (partly in colors). Price in cloth, $2.50. P. Blakiston, Son & Co., Publishers, 1012 Walnut Street, Philadelphia.

A large number of general practitioners realize the advantages that a thorough knowledge of the use of the microscope would be in making a correct diagnosis, and many of them would be glad to avail themselves of this valuable aid, but they have not the time to, and can not afford the expense of going to a medical school to take a special course in microscopy. The idea of gaining a perfect knowledge of the use and application of the microscope at home, has not occurred to most of them. But with Dr. Reeves' little book as his instructor, it would not take even the mediocre very long to become master of this branch of the medical science. In this book Dr. Reeves gives plain and thorough instructions for the selection of the microscopic outfit, how to work with the instrument, preparation of animal tissues, fixing and hardening of tissues, cutting sections, fixing sections immediately on the slide, staining and finishing, and formularies for preparing stains.

He also devotes a chapter to bacteriology, one to the examination of neoplasms, one to urinary examinations, one to the examination of the blood, one to tbe sputum, one to fecal discharges, one to mucous cylinders, one to micro-organisms of the skin, and one on micro-organisms in foods and drinks.

Quite a number of the illustrations are in colors, and greatly enhance the beauty and merit of the book. H.

---

THE MEDICAL NEWS VISITING LIST for 1895. Weekly (dated, for 30 patients); Monthly undated, for 120 patients per month); Perpetual (undated, for 30 patients weekly per year); and Perpetual (undated, for 60 patients weekly per year). The first three styles contain 32 pages of data and 160 pages of blanks. The 60-Patient Perpetual consists of 256 pages of blanks. Each style is one wallet-shaped book, with pocket, pencil and rubber. Seal Grain Leather, $1.25. Philadelphia: Lea Bros. & Co., 1894.

The Medical News Visiting List for 1895 has been thoroughly revised and brought up to date in every respect. The text portion (32 pages) contains the most useful data for the physician and surgeon, including an alphabetical table of diseases, with the most approved remedies, and a table of doses. It also contains sections on examination of urine, artificial respiration, incompatibles, poisons and antidotes, diagnostic table of eruptive fevers, and the ligation of arteries. The classified blanks (160

pages) are arrangrd to hold records of all kinds of professional work, with memoranda and accounts. Much care has been bestowed upon the mechanical execution of the book, and in quality of paper and in strength and beauty of binding, nothing seems to be left wanting. When desired, a Ready Reference Thumb-letter Index is furnished, which will save manifold its small cost (25 cents) in the economy of time effected during the year. In its several styles the Medical News Visiting List adapts itself to any system of keeping professional accounts. In short, every need of the physician seems to have been anticipated in this invaluable pocket companion.

---

ESSENTIALS OF NERVOUS DISEASES AND INSANITY: Their Symptoms and Treatment. A Manual for Students and Practitioners. By John C. Shaw, M. D., Clinical Professor of Diseases of the Mind and Nervous System, Long Island Hospital Medical School; Consulting Neurologist to St. Catherine's Hospital and Long Island College Hospital; formerly Medical Superintendent King's County Insane Asylum. Second edition, revised. Forty-eight original illustrations, mostly selected from the author's private practice. Price, in cloth, $1.00. W. B. Saunders, publisher, 925 Walnut street, Philadelphia. 1894.

As its name indicates—this little work of one hundred and ninety-four pages, is only a manual, and is not intended to take the place of the larger and more complete works on the same subjects. It is intended to be used especially as a primer for advanced students.

The various diseases of the nervous system are treated of in a concise way, and the essential facts, as at present understood, bearing on their etiology, pathology, etc., are clearly stated.

The scope of the work would not permit the author to include all of the diseases coming under the nomenclature of diseases of the nervous system and insanity, but he has exercised good judgment in selecting those frequently met with in general practice. The arrangement and descriptions are the most simple that could be made.

